# Control of regional decidualization in implantation: Role of FoxM1 downstream of Hoxa10 and cyclin D3

**DOI:** 10.1038/srep13863

**Published:** 2015-09-09

**Authors:** Fei Gao, Fenghua Bian, Xinghong Ma, Vladimir V. Kalinichenko, Sanjoy K. Das

**Affiliations:** 1 Division of Reproductive Sciences, Cincinnati Children’s Hospital Medical Center, University of Cincinnati College of Medicine, Cincinnati, OH 45229, USA; 2 Perinatal Institute, Cincinnati Children’s Hospital Medical Center, University of Cincinnati College of Medicine, Cincinnati, OH 45229, USA; 3 Division of Pulmonary Biology, Cincinnati Children’s Hospital Medical Center, University of Cincinnati College of Medicine, Cincinnati, OH 45229, USA; 4 College of Life Science, Northeast Agricultural University, Harbin, China.

## Abstract

Appropriate regulation of regional uterine stromal cell decidualization in implantation, at the mesometrial triangle and secondary decidual zone (SDZ) locations, is critical for successful pregnancy, although the regulatory mechanisms remain poorly understood. In this regard, the available animal models that would specifically allow mechanistic analysis of site-specific decidualization are strikingly limited. Our study found that heightened expression of FoxM1, a Forkhead box transcription factor, is regulated during decidualization, and its conditional deletion in mice reveals failure of implantation with regional decidualization defects such as a much smaller mesometrial decidua with enlarged SDZ. Analysis of cell cycle progression during decidualization both *in vivo* and *in vitro* demonstrates that the loss of *FoxM1* elicits diploid cell deficiency with enhanced arrests prior to mitosis and concomitant upregulation of polyploidy. We further showed that Hoxa10 and cyclin D3, two decidual markers, control transcriptional regulation and intra-nuclear protein translocation of FoxM1 in polyploid cells, respectively. Overall, we suggest that proper regional decidualization and polyploidy development requires FoxM1 signaling downstream of Hoxa10 and cyclin D3.

Uterine stromal cells undergo transformation into morphologically and functionally distinct cells called decidual cells (decidualization), which occurs in women during the secretory phase of the menstrual cycle as well as in pregnancy; in rodents, this process only occurs during pregnancy. The onset of decidualization following embryo implantation is essential for successful pregnancy^1^,^2^. In the receptive uterus on day 4 (D4) of pregnancy (D1 = vaginal plug) in mice, uterine stromal cells experience proliferation under the coordinated control of both ovarian estrogen and progesterone. However, following embryonic attachment to the uterine luminal epithelium, which occurs at 24:00 h on D4, stromal cells proximally surrounding the implantation chamber exhibit rapid proliferation and spreading. By D5 morning, these cells can be found throughout the stromal bed. The first sign of stromal differentiation, forming of the primary decidual zone (PDZ), occurs in the first few layers of cells at the antimesometrial location of the implantation site (IS) in the afternoon on D5^3^,^4^. PDZ is avascular and epithelioid in nature^5^. From D6 through D8, stromal cells next to the PDZ continue to proliferate and differentiate to form polyploidy in the secondary decidual zone (SDZ), which develops both at the lateral and antimesometrial locations of the IS. In contrast to SDZ development, mesometrial stromal cells continue to proliferate and differentiate to form the non-polyploid decidual zone, a presumptive site for placentation.

Decidual polyploidization is a hallmark of terminally differentiated cells and has been well characterized in rodents^3^,^4^,^6^,^7^,^8^,^9^ and recently recognized in humans [Hirota Y and Dey SK (unpublished observations)]. These cells undergo endoreduplication cycle to develop as giant mono- or bi-nuclear cells with multiple copies of chromosomes^3^,^4^,^6^,^7^,^8^,^9^ and possess increased mitochondrial activity^6^. The loss of decidual polyploidy in association with pregnancy failure by mid-gestation has been reported in *Dedd* null mice^10^.

Uterine decidualization in implantation is believed to be regulated through complex signaling mechanisms that involve homeobox transcription factors, cell-cycle genes, cytokines, growth factors, lipid mediators, and other regulatory molecules^1^,^2^,^11^,^12^. However, there remains a major gap in understanding the mechanisms that control regional (mesometrial vs. antimesometrial) decidual development in implantation. The homeobox transcription factor Hoxa10 has been shown to play an important role in directing proper regional decidual development^11^,^13^. It has been shown the *Hoxa10* null mutation in mice produces a lack of uterine stromal cell proliferation in response to progesterone and consequentially results in the failure of proper decidua growth^14^,^15^,^16^. Consistently, cyclin D3—a G1 phase cell cycle regulator for stromal cell proliferation, differentiation, and polyploidy development^3^,^4^,^17^—exhibits severe downregulation of expression during decidual progression in *Hoxa10* null mice^13^,^17^. Moreover, studies have shown that adenovirus-driven overexpression of cyclin D3 at the site of implantation improves decidualization defects in *Hoxa10*^*−/−*^ mice^18^, indicating cyclin D3 plays an important role downstream of Hoxa10 during decidualization.

FoxM1, a member of the large family of Forkhead box transcription factors, is highly expressed in proliferating cells and plays pivotal roles in DNA replication and mitosis through modulation of diverse regulatory genes involved in transitions between G1-S and G2-M phases of the cell cycle^19^. It has been well recognized that FoxM1 is robustly expressed by oncogenic signals in almost all types of malignant tumor tissues and cancer cell lines^20^, and is highly expressed in a broad range of tissues during embryo development^19^,^21^. However, its expression is found in few normal adult tissues^19^. Our findings as reported here have provided new evidence that FoxM1 is expressed and regulated in the early post-implantation uteri during decidualization. By utilizing genetic knockout mouse models, we have provided novel evidence that FoxM1 is regulated during stromal cell decidualization and uterine conditional deletion of *FoxM1* reveals regional decidualization defects via impaired stromal cell mitosis and aberrantly upregulated polyploidy at the site of implantation. Further, we showed that FoxM1 is regulated at the transcriptional level by Hoxa10 and in its intra-nuclear protein localization by cyclin D3.

## Results

### FoxM1 is regulated during uterine stromal cell proliferation and differentiation for decidualization

**Figure 1:**
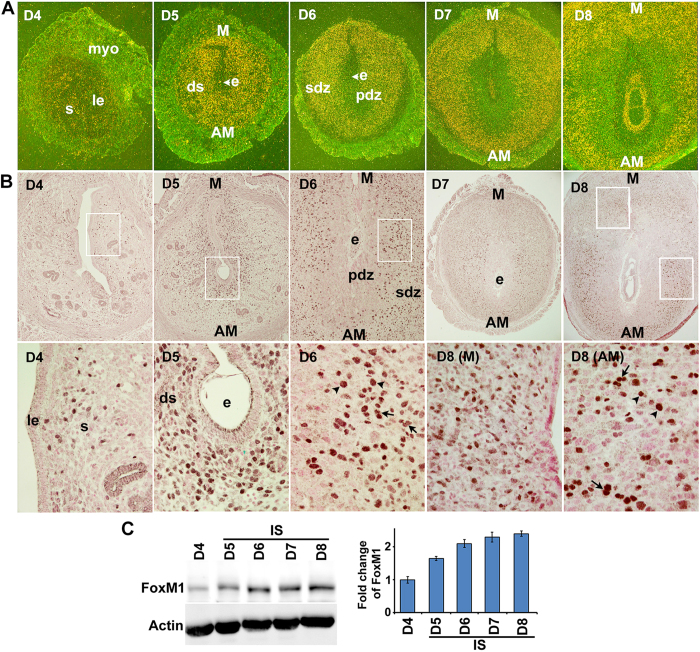
FoxM1 expression in peri-implantation uteri. (**A**) *In situ* hybridization of *FoxM1* expression on D4-8 of pregnancy. le, luminal epithelium; s, stroma; myo, myometrium; e, embryo; ds, decidualizing stroma; pdz, primary decidual zone; sdz, secondary decidual zone; M, mesometrial; AM, antimesometrial. The magnifications are at 40X. (**B**) Immunostaining of FoxM1 on D4-8 of pregnancy. Dark brown staining indicates positive signals; nuclei are counterstained with fast red. le, luminal epithelium; s, stroma; e, embryo; ds, decidualizing stroma; pdz, primary decidual zone; sdz, secondary decidual zone; M, mesometrial; AM, antimesometrial. Arrows and arrowheads indicate mono and bi-nucleated polyploid cells, respectively. Lower panels show magnified images for selected areas shown in corresponding upper panels. The magnifications in the upper panels are at 100X (D4-6) and 40X (D7-8), while in the lower panels are at 400X. (**C**) Western blotting for FoxM1 analysis. Bar diagram shows relative levels of expression after normalization with actin.

To better understand the role of uterine FoxM1 during the periimplantation period, we examined the spatiotemporal expression of FoxM1 mRNA and protein on the receptive day (D4) and postimplantation uteri on D5-8. Our *in situ* hybridization results show a moderate expression with scattered distribution within the endometrial stroma on D4. In contrast, a heightened expression was noted in decidualizing stromal cells throughout the endometrium at the IS on D5 (Fig. 1A). However, at the IS on D6-8, *FoxM1* expression was predominantly localized in the SDZ, as well as in the M-polar decidual bed (Fig. 1A), and this pattern of expression was enhanced with the progression of pregnancy. Cell-specific localization of FoxM1 immunoreactive protein was consistent with that of mRNAs (Fig. 1B). More specifically, nuclear FoxM1 was noted with a scattered distribution in the stromal bed on D4 (Fig. 1B). On D5, nuclear signals were distinctly upregulated in the sub-luminal stromal cells at the IS (Fig. 1B). However, on D6–8, both decidual polyploid (in SDZ) and non-polyploid (in M-polar bed) cells were positive for nuclear FoxM1 localization (Fig. 1B). Consistent with the above results, further quantitative analyses of FoxM1 by western blotting also revealed an increased gradual accumulation of protein levels at the IS on D5-8, while D4 also had low levels (Fig. 1C).

Since FoxM1 is critical for cell cycle progression, we next analyzed FoxM1 expression by combined immunofluorescence with cell cycle phase-specific markers, incorporating BrdU (S-phase for DNA synthesis) and phosphorylated histone H3 (pHH3) (M-phase for mitosis) in periimplantation uterine sections on D4 as well as at D5-D8 IS. In general, FoxM1 positive stromal/decidual cells were co-localized either with BrdU or pHH3 on D4-D8 of pregnancy, indicating that FoxM1 indeed plays a role in cell cycle progression during the S- and M-phases (Supplementary Fig. S1A, representative data on D6-IS is shown for mesometrial and antimesometrial locations). However, quantitative analyses of localization showed that although dual positive FoxM1/BrdU cells were significantly upregulated at the mesometrial location from D5 through D8, there was a slight decline in levels on D7 and D8 (Supplementary Fig. S1B). Similarly, FoxM1/pHH3 cells were also upregulated between D6 through D8, but with a decline on D8 (Supplementary Fig. S1B). In contrast, at the SDZ for both lateral (L) and antimesometrial (AM) locations, FoxM1/BrdU double labelled cells were significantly increased from D6 through D8 with a peak on D6, while FoxM1/pHH3 cells did not reveal any change (Supplementary Fig. S1B) This suggests that FoxM1 positive SDZ cells may lack mitotic progression despite an increase in S-phase activity, a condition that is conducive for polyploidy development^3^,^4^. Overall, FoxM1 expression is closely associated with stromal cell proliferation and differentiation, including terminal differentiation with polyploidy development.

### Uterine deletion of *FoxM1* leads to decidualization defects in early pregnancy

**Figure 2:**
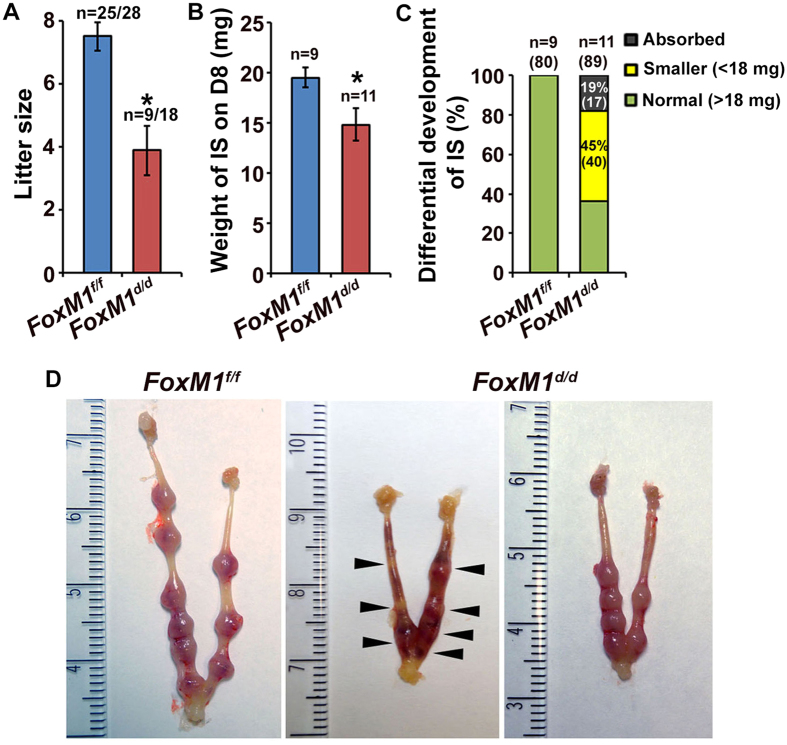
Uterine deletion of *FoxM1* results in female subfertility with loss of embryo implantation during decidualization. (**A**) Pregnancy outcome in null (*FoxM1*^*d/d*^) and control (*FoxM1*^*f/f*^) littermates after mating with fertile males. n, indicates number of females with pups out of total plug-positive females for each genotype. Results shown as Mean ± SEM. *Significantly different (p < 0.05). (**B**) Weight of IS on D8 for null and control littermates. n, indicates number of females with IS for each genotype. Results shown as Mean ± SEM. (**C**) Developmental competence of IS between null and control on D8. n, indicates number of pregnant females, with a total number of IS (in parentheses) for each group. The percent with total number of IS for each category (absorbed, smaller, and normal) also indicated by color codes. (**D**) Uterine IS morphology for *FoxM1*^*d/d*^ and *FoxM1*^*f/f*^ mice is shown on D8. Arrowheads point to resorption sites.

To explore the role of uterine FoxM1 in early pregnancy, we used *PR*-Cre mice to specifically delete *FoxM1* in the uterus. Our analyses clearly revealed deletion of *FoxM1* in null (*FoxM1*^*d/d*^) as compared to control (*FoxM1*^*f/f*^) at the IS on D8, as examined by qRT-PCR (Supplementary Fig. S2A). We next analyzed pregnancy outcome between null and control mice. We observed that the homozygous deletion of *FoxM1* causes severe subfertility, as compared to control (Fig. 2A). These results were consistent with a significant decrease in average IS weight on D8 for null mice, as compared to control (Fig. 2B). However, based on uterine gross morphology and distribution of D8 IS weight, we arbitrarily divided into three parts for null: ∼19% (17 out of 89) of IS were primarily absorbed and darkly bloody while another ∼45% (40 out of 89) of IS were much smaller (<18 mg) without showing any sign of resorption compared to normal (>18 mg) (Fig. 2C,D), indicating that loss of *FoxM1* differentially affects IS’s developmental progression during decidualization. Consistent with these observations, after the induction of artificial decidualization, we also noted that only 37.5% of null mice were responsive to the onset of decidualization, but with a reduction in response by ∼3-fold, as compared to control (Supplementary Fig. S3A–C). In addition, the above defects were not associated with any disturbances in ovarian hormone levels in serum (Supplementary Fig. S2B) or ovarian corpora luteal structures (Supplementary Fig. S2C). Whereas only ovarian granulosa cells in the preantral/antral follicles for control mice had detectable FoxM1 expression and this was not affected in null mice (Supplementary Fig. S2C); this was consistent with the findings of a prior study that found only corpus luteal Cre-expression using a *Pgr*-driven promoter^22^. Overall, our results suggest that insufficient decidual progression in implantation is a primary cause for early pregnancy defects in *FoxM1*^*d/d*^ mice.

### *FoxM1* deficiency leads to mesometrial decidua shrinkage with increased SDZ and polyploidy development in early pregnancy

**Figure 3:**
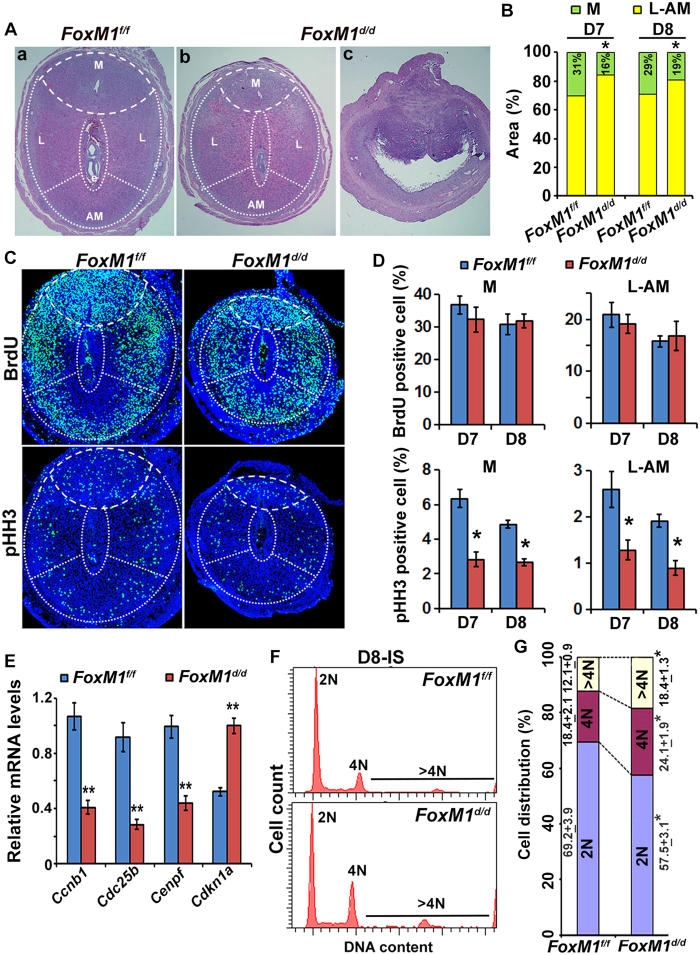
Defective regional decidualization with abnormal cell cycle activity results from the loss of uterine *FoxM1*. (**A**) (a,b), Histological analyses by hematoxylin and eosin staining of D8-IS between null (*FoxM1*^*d/d*^) and control (*FoxM1*^*f/f*^) mice. Mesometrial (M), lateral (L) and antimesometrial (AM) locations are demarcated by broken lines. (c), A representative IS shows signs of resorption for null mice. (**B**) Comparison of developmental area (%) for M and SDZ (L+AM) locations on D7-8 IS between null and control mice. *Significantly different (p < 0.001) for corresponding M or SDZ locations between null and control on particular day of pregnancy. (**C**) Immunofluorescence analyses of BrdU and pHH3 at D7-IS between null and control. (**D**) Quantitative analyses of BrdU or pHH3 positively stained cells (%) in M and SDZ locations of D7-8 IS between null and control mice. *Significantly different (p < 0.001) between null vs. control. (**E**) Quantitative RT-PCR analyses of cell cycle regulatory genes [*Ccnb1*, *Cdc25b, Cenpf, and Cdkn1a* (*p21*)] at D7-IS for control and null mice. **Significantly different (p < 0.001) between null vs. control. (**F**) A representative flow cytometric analysis of cell cycle distribution based on DNA content for decidual cells isolated on D8-IS between null vs. control. (**G**) Cellular distribution (%) for 2N, 4N, and >4N populations at the D8-IS between null vs. control. *Significantly different (p < 0.001) for corresponding groups between control vs null mice.

To determine the cause for early implantation defects, we compared implantation progression histologically on serial sections between *FoxM1* null and control mice. As expected, null ISs were found to be in the process of resorption, as clearly revealed by embryo loss and collapse of decidua structure (Fig. 3Ac). However, the analysis of partially compromised null sites revealed that the mesometrial decidual bed was significantly smaller with a concomitant increase in the SDZ (lateral and antimesometrial regions) than control on D8 (Fig. 3Aa,b,B), as well as on D7 (Fig. 3B). Embryonic growth was also significantly smaller for null as compared to control (Fig. 3Aa,b). Despite compromised decidualization, gene expression for stromal cell decidualization (*Bmp2* and *Hoxa-10*)^12^,^16^, local vascularization (*Ang2* and *Ptgs2*)^23^,^24^, and polyploidy development (*Ccnd3* and *Trp53*)^3^,^25^,^26^ was not affected at the IS on D7 or D8 between control and null mice, indicating FoxM1 is not involved with the regulation of these molecular events. Overall, *FoxM1* essentially controls proper regional decidualization without affecting many implantation specific genes.

We next evaluated whether cell cycle activity was affected by the loss of *FoxM1* during decidualization. Analyses of BrdU (for S-phase) and pHH3 (for M-phase) at the IS on D7 (Fig. 3C,D) and D8 (Fig. 3D) revealed that although accumulation of BrdU positive cells was not affected, pHH3 labelled cells at the mesometrial and SDZ (L+M) locations were significantly reduced in null as compared to control, indicating loss of *FoxM1* crucially affects cell cycle progression in M-phase, but not S-phase, during decidual progression. Consistently, quantitative RT-PCR analyses demonstrated that expression of G2/M phase regulators *Ccnb1*, *Cdc25b,* and *Cenpf* were significantly downregulated, whereas the cyclin-dependent kinase inhibitor/differentiation regulator *Cdkn1a* (*p21*) was upregulated in null as compared to control (Fig. 3E). Interestingly, all aforementioned mitotic genes have been known to act as transcriptional targets upregulated by FoxM1^27^,^28^,^29^. P21 has been found to be downregulated by FoxM1 in other cell types^29^,^30^. However, the expression of several G1/S phase regulators (*Ccnd3*, *Ccna1*, *Ccna2*, *Ccne1*, *Ccne2*, *Cdc25a*, *cdk4*, *cdk6, cdk2*, *E2f1*, *Cks1b*, *Skp2* and *Gas1*), other G2/M phase regulators (*Ccnb2*, *cdk1*, *Plk*, *Aurkb*, *Birc5* and *Nek2*), and cdk inhibitors (*cdkn1b* and *cdkn1c*) were not affected between the mice, although many of these genes have been shown as FoxM1 transcriptional targets in other cell types^30^.

The arrest of decidual cell development prior to entering M-phase is crucially linked with the onset of polyploidy^3^,^4^; thus, we next analyzed ploidy levels by flow cytometric analyses of DNA content between null and control mice during decidualization. Our analyses revealed that 2N cells were significantly decreased with a concomitant enhancement of 4N and >4N cells within the decidual bed for null mice as compared to control on D8-IS (Fig. 3F,G), suggesting FoxM1 is critical for the balanced progression of diploid cell and polyploidy levels during decidualization. This phenomenon was also consistent with our observation of increased bi-nuclear polyploid cells in the SDZ for null mice as compared to control on D8 (Supplementary Fig. S4). Overall, the proper development of mesometrial decidua and SDZ at the site of implantation necessitates uterine *FoxM1,* primarily to appropriately control diploid vs. polyploid decidual cell status during decidualization.

### *FoxM1* is critical to appropriately balance mitosis and polyploidy development during stromal cell decidualization *in vitro*

**Figure 4:**
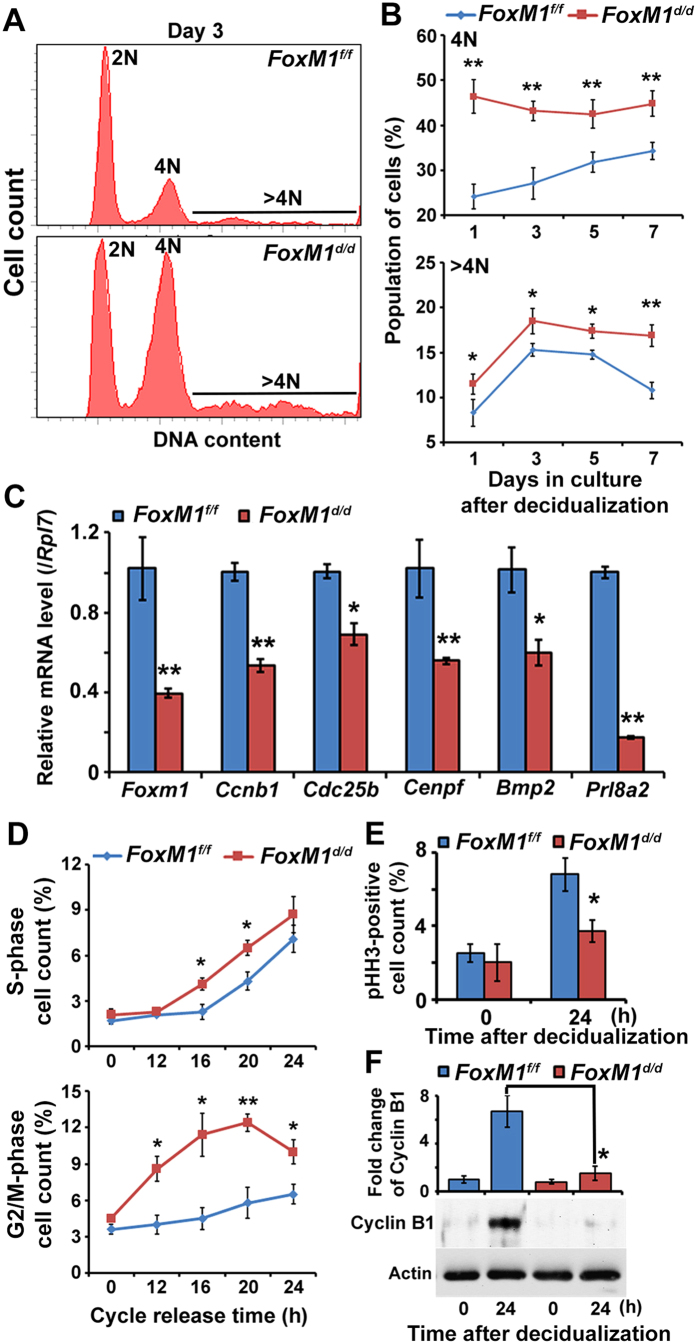
*FoxM1* deficiency in stromal cells leads to enhanced cell cycle arrest prior to mitosis with aberrantly upregulated polyploidy during decidualization *in vitro*. (**A**) A representative flow cytometric analysis of cell cycle distribution based on DNA content for cells after *in vitro* decidualization for 3-days between null (*FoxM1*^*d/d*^) vs. control (*FoxM1*^*f/f*^) stromal cells. (**B**) Cellular distribution (%) for 4N and >4N populations after *in vitro* decidualization at indicated days (1–7 days) between null vs. control cells. **p < 0.001 and *p < 0.05. (**C**) Quantitative RT-PCR analyses of *FoxM1, Ccnb1*, *Cdc25b, Cenpf, Bmp2, and Prl8a2* after *in vitro* decidualization for 3 days between null and control cells. **p < 0.001 and *p < 0.05. (**D**) Cell cycle phase-specific count (%) in S and G2/M after the release in cell cycle for proliferation *in vitro* at indicated times (0–24 h) between null and control cells. **p < 0.001 and *p < 0.05. E. Quantitative analysis of pHH3-positive cell count (%) after *in vitro* decidualization at indicated times (0 and 24 h) between null and control cells. *p < 0.05. F. Analysis of Cyclin B1 protein after *in vitro* decidualization at indicated times (0 and 24 h) between null and control cells. *p < 0.001.

To analyze the mechanism for aberrant polyploidy development in *FoxM1*^*d/d*^ mice, we examined cell cycle progression during stromal cell decidualization *in vitro*. Following the induction of decidualization^31^, our analysis revealed that *FoxM1* null stromal cells, as opposed to control, were able to show increased accumulation of 4N and >4N (polyploid) cells during days 1–7 in culture (Fig. 4A,B), indicating *FoxM1-*deficiency results in aberrant upregulation of cells with polyploidy. Meanwhile, 2N cells decreased in null compared to control. *FoxM1* null cells exhibited aberrant downregulation of expression for G2/M-phase cell cycle regulators (*Ccnb1*, *Cdc25b,* and *Cenpf*) and markers of decidualization (*Bmp2* and *Prl8a2*), as compared to control (Fig. 4C), indicating *FoxM1-*deficient cells probably suffer from cell cycle arrest during G2/M with defective decidual progression. Because decidualization process depends on simultaneous progression of proliferation and differentiation, to specifically define FoxM1 regulation in proliferation, synchronized FoxM1 null or control stromal cells were reactivated in order to re-enter the cell cycle by serum for 0–24 h in culture. Our analyses revealed that despite significantly enhanced accumulation of *FoxM1* null cells compared to control, either in S (at 16–20 h) or G2/M (at 12–24 h) (Fig. 4D), the level of pHH3 (M-phase marker)-positive cells (Fig. 4E) and expression of cyclin B1 (M-phase regulator) (Fig. 4F) were strikingly lower at 24 h post reactivation for null cells compared to control, indicating *FoxM1* deficiency results in cell cycle arrest prior to the M phase. Our analysis of FoxM1 in control cells revealed that the expression was induced both at mRNA and protein levels, during cell cycle phase progression, particularly at 12–24 h following the cycle release (Supplementary Fig. S5). In addition, FoxM1 expression by immunofluorescence localization did not reveal any obvious intracellular trafficking, since the expression remained in nuclear location during this period. Overall, the loss of *FoxM1* in stromal cells leads to enhanced cell cycle arrest prior to mitosis with aberrantly upregulated polyploidy during decidualization *in vitro*.

### *FoxM1* expression is regulated at the transcription level by Hoxa10 during decidualization

**Figure 5:**
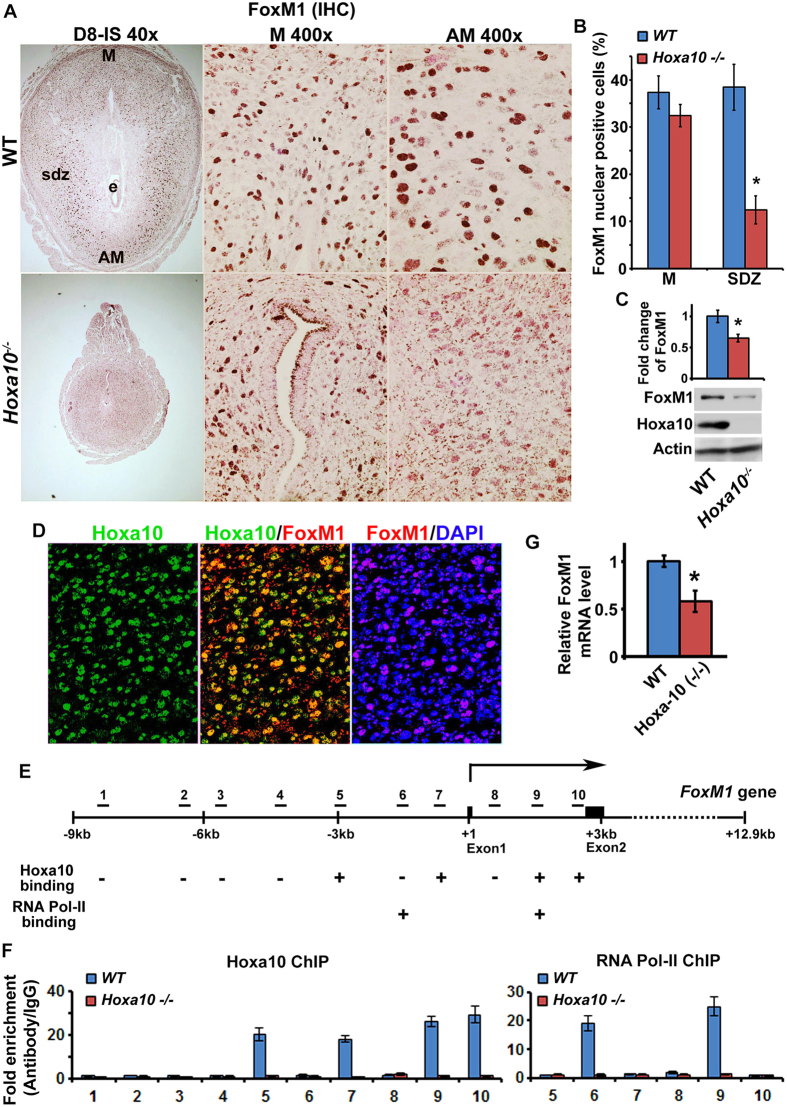
Hoxa10 regulates FoxM1 in SDZ cells via a transcriptional mechanism. (**A**) Immunohistochemical analysis of FoxM1 at IS on D8 between wild-type (WT) and *Hoxa10*^*−/−*^ mice. High magnification pictures (at 400X) are shown for mesometrial (M) and antimesometrial (AM) locations. Note: Data show reduced expression of FoxM1 at the SDZ location (lateral or antimesometrial) in *Hoxa10*^*−/−*^ compared to that of WT. e, embryo; sdz, secondary decidual zone; M, mesometrial; AM, antimesometrial. (**B**) Quantitation of nuclear FoxM1 positive cells in M and SDZ locations at IS on D8 between WT and *Hoxa10* null mice. (**C**) Western blot analyses of FoxM1, Hoxa10, and actin at IS on D8 between WT and *Hoxa10* null mice. Quantitative analysis of FoxM1 expression shows in the Bar plot. (**D**) Dual immunofluorescence analysis of Hoxa10 (green) and FoxM1 (red) at the SDZ on D8-IS. DAPI was used for nuclear staining. (**E**) Diagrammatic illustration of putative Hoxa10 binding regions (1–10) in 5’-flanking, exon 1, and intron 1 of *FoxM1* gene. “+” or “–” indicates actual detection of positive or negative binding of Hoxa10 or RNA Pol-II. (**F**) Quantitative ChIP-PCR analysis of Hoxa10 or RNA Pol II binding for WT and *Hoxa10* null in deciduoma tissues on D7, as described in Materials and Methods. (**G**) Analysis of *FoxM1* expression in deciduoma tissues on D7 by quantitative RT-PCR between WT and *Hoxa10* null mice, after normalization with *Rpl7*, a housekeeping gene.

Hoxa10 is a major regulator of decidualization in early pregnancy^11^,^13^,^14^. To determine whether FoxM1 is regulated downstream of Hoxa10 during decidualization, we examined FoxM1 expression at IS between *Hoxa10*^*−/−*^ mice as compared to wild-type (WT) on D8. Immunohistochemical analyses show that nuclear FoxM1 expression at the SDZ was significantly reduced in *Hoxa10*^*−/−*^ mice as compared to WT (Fig. 5A,B). In contrast, mesometrial FoxM1 expression was comparable between WT and *Hoxa10* null mice (Fig. 5A,B). Consistently, quantitation of protein levels by western blotting also revealed downregulation of FoxM1 expression in *Hoxa10* null, as compared to WT (Fig. 5C). Since Hoxa10 is a transcriptional factor, we next examined whether the above regulation of *FoxM1* expression is directly controlled by Hoxa10. To examine this notion, we first evaluated dual expression analysis of FoxM1 and Hoxa10 at IS on D8 for WT. Based on dual immunofluorescence studies, we observed that FoxM1 expression was co-localized with Hoxa10 at the SDZ (Fig. 5D). Next, to analyze a possibility for direct regulation of *FoxM1* by Hoxa10 during decidualization, we examined the relationship by chromatin immunoprecipitation (ChIP) studies. We identified several Hoxa10 binding consensus sequences by computational analysis of the 10 kb region for the 5′-flanking, first exon, and first intron of the *FoxM1* gene (Fig. 5E). Our analyses of Hoxa10 ChIP followed by qPCR showed that several distinct regions within the promoter and gene body are responsible for Hoxa10 binding, since they are undetected in *Hoxa10*^*−/−*^ mice (Fig. 5F). These results are consistent with analyses for RNA polymerase II recruitment by ChIP (Fig. 5F) and the status of FoxM1 mRNAs by RT and qPCR (Fig. 5G) between WT and *Hoxa10* null mice. Overall, these results suggest that Hoxa10 is necessary to control FoxM1 expression in SDZ cells via transcriptional mechanism.

### Intra-nuclear translocation of FoxM1 protein is regulated by cyclin D3 via the cyclin-dependent kinase pathway

**Figure 6:**
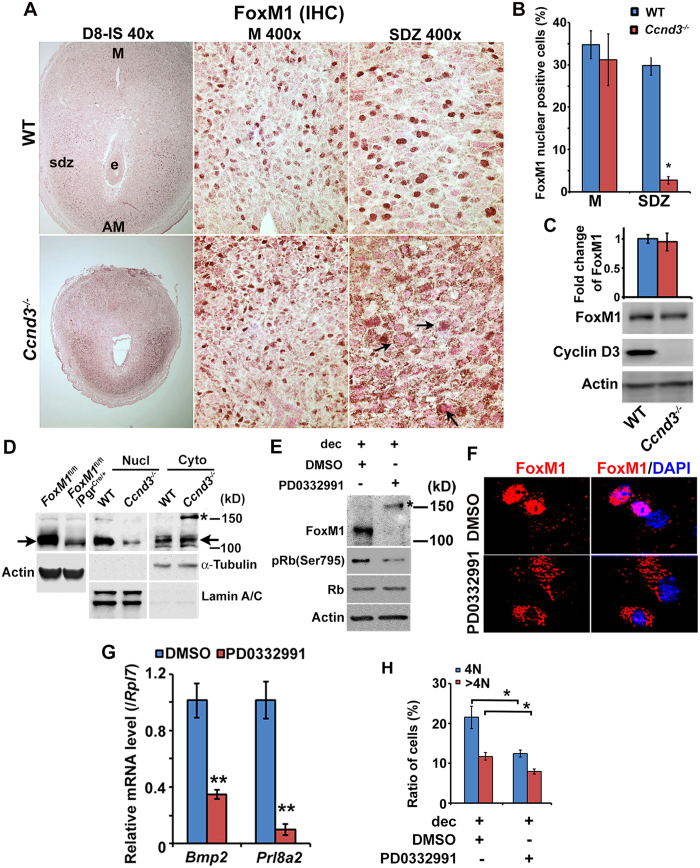
Cyclin D3 regulates nuclear FoxM1 localization in SDZ cells via cyclin-dependent kinase mechanism. (**A**) Immunohistochemical analysis of FoxM1 at D8-IS between WT and *Ccnd3*^*−/−*^ mice. High magnification pictures (at 400X) are shown for mesometrial (M) and antimesometrial (AM) locations. Note: Cyclin D3 deficiency causes loss of nuclear FoxM1 at SDZ location (lateral or antimesometrial) as compared to WT. e, embryo; sdz, secondary decidual zone; M, mesometrial; AM, antimesometrial. Arrows indicate cytoplasmic accumulation of immunostained signals. (**B**) Quantitation of nuclear FoxM1 positive cells in M and SDZ locations at D8-IS between WT and *Ccnd3* null mice. (**C**) Western blot analyses of FoxM1, cyclin D3, and actin at IS on D8 between WT and *Ccnd3* null mice. Quantitative analysis of FoxM1 expression shows in the Bar plot. (**D**) Western blot analyses of FoxM1, α-Tubulin and Lamin A/C in nuclear (Nucl) and cytoplasmic (Cyto) fractions of SDZ between WT and *Ccnd3*^*−/−*^ mice. The expression of α-Tubulin and Lamin A/C was used to judge purity of cytoplasmic and nuclear fractions, respectively. Western blot analyses for *FoxM1* null vs. control cells show the specificity of antibody detected bands for comparison. Actin was used as control. Note: The level of FoxM1 was downregulated in nuclear fraction, but concomitantly upregulated in cytoplasmic fraction with detection of an additional band ∼150 kDa [shown by asterisk (*)], an indication of protein modification. FoxM1 full-length protein size ∼109 kDa is shown by arrows. E. Western blot analyses of FoxM1, pRb(Ser795), Rb, and Actin during *in vitro* decidualization after inhibition of Cdk4/6 activity by PD0332991 (10 μM), as compared to control (DMSO). (**F**) Immunofluorescence analyses of FoxM1 localization after addition of PD0332991 or DMSO during *in vitro* decidualization. (**G**) Quantitative RT-PCR analyses of decidual marker genes (*Bmp2* and *Prl8a2*). **Significantly different (p < 0.001) between the treated groups. (**H**) Flow cytometric analysis cell cycle distribution for 4N and >4N cells during *in vitro* decidualization after addition of PD0332991 or DMSO. *Significantly different (p < 0.05) between the compared groups.

Cyclin D3 plays a major role in cell cycle regulation during stromal cell decidualization^3^,^4^,^17^, and since FoxM1 also plays roles in cell cycle events^19^, we next wanted whether cyclin D3 controls FoxM1 during decidualization. FoxM1 localization at IS on D8 between *Ccnd3* null and WT mice was examined by immunohistochemical analyses. Results showed that although nuclear localization of FoxM1 protein at the mesometrial decidual cells was similar between mice, nuclear positivity of FoxM1 in polyploid cells at the SDZ was significantly lower along with cytoplasmic accumulation (shown by arrows) in *Ccnd3*^*−/−*^ as compared to WT (Fig. 6A,B). However, quantitative analysis of FoxM1 protein levels by western blotting did not reveal any change between mice (Fig. 6C). Furthermore, fractionation of SDZ cells into nuclear and cytoplasmic protein extracts followed by western blotting analyses revealed that the level of FoxM1 was downregulated in nuclear fraction, but concomitantly upregulated in cytoplasmic fraction with detection of an additional band ∼150 kDa [shown by asterisk (*)], an indication of protein modification, in *Ccnd3*^*−/−*^ as compared to WT (Fig. 6D). In addition, the inclusion of western blot results for *FoxM1* null vs. control cells show the specificity of antibody detected bands for comparison (Fig. 6D). FoxM1 full-length protein size ∼109 kDa is shown by arrows (Fig. 6D). The purity of the above fractions was apparently normal, as judged by the analyses of lamin A/C and α-tubulin for corresponding nuclear and cytoplasmic proteins (Fig. 6D). In order to evaluate the mechanism for this regulation, we next used *in vitro* uterine stromal cell decidualization^31^ following the inhibition of cdk4/cdk6 kinase activity. The application of an inhibitor (PD0332991) as compared to control (DMSO as a vehicle) caused inhibition of FoxM1 nuclear accumulation, yet a high molecular protein band was still clearly visible [shown by asterisk (*)] (Fig. 6E). These results were also consistent with immunofluorescence studies that found a loss of nuclear FoxM1 localization (Fig. 6F), downregulation of decidualization marker expression (Fig. 6G), and lesser accumulation of 4N and >4N (polyploid) cell populations (Fig. 6H), with the application of an inhibitor versus the control. Overall, these results suggest that cyclin D3 crucially controls nuclear localization of FoxM1 with polyploidy in a cdk4/6 kinase dependent manner.

## Discussion

**Figure 7:**
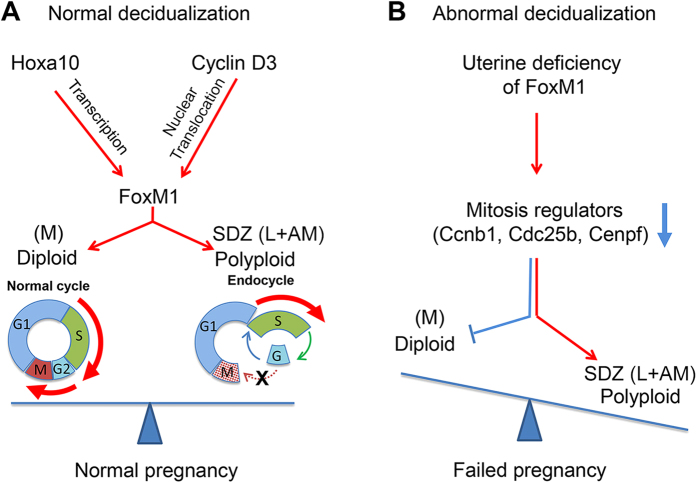
A proposed model depicting the role of FoxM1, downstream of Hoxa10 or cyclin D3, in mediating appropriate control for stromal cell diploid vs polyploid status during decidualization. Hoxa10 plays a major role in decidualization primarily through upregulation of cyclin D3 in decidualizing stromal cells and also exerts critical regulation in regional decidualization in implantation; mesometrial (M) vs. SDZ development. Here, we provide evidence that the expression of FoxM1 is regulated during decidualization and is controlled by Hoxa10 for transcription and by cyclin D3, a target of Hoxa10, for nuclear FoxM1 protein localization (**A**). Based on FoxM1 deletion studies, we noted that FoxM1 is critical for normal female fertility, primarily to control appropriate regional progression of decidualization [M vs SDZ development]. The loss of FoxM1 leads to the failure of implantation with decidualization defects; smaller decidual in M-bed, due to 2N cell deficiency with increased arrests prior to mitosis and a bigger SDZ with aberrantly upregulated polyploidy, indicating FoxM1 is critical to appropriately balance diploid vs. polyploid cell status during decidualization (**B**).

The successful regional decidualization in implantation has been recognized by development of the diploid mesometrial decidual triangle (a site for placentation), together with polyploid SDZ in antimesometrial location. Although polyploidization occurs in terminally differentiated decidual cells and its development during implantation has shown to be critical for pregnancy^10^,^26^, the underlying regulatory mechanisms for establishing these two regions remain poorly studied. The highlight of the present investigation is that FoxM1 is critical for controlling regional (mesometrial vs. antimesometrial) development in implantation through a finely tuned, cell cycle-based regulatory balance between diploid and polyploid decidual cell status (Fig. 7). Furthermore, we showed that two decidual markers - Hoxa10 and cyclin D3 - control respective FoxM1 gene transcription and protein translocation to polyploid cell nuclei. Uterine conditional deletion of *FoxM1* causes a reduction in the mesometrial decidual area, thereby lowering diploid cell content primarily due to increased arrests prior to mitosis and enhancing the commitment for polyploidy and thus producing aberrant expansion of the SDZ.

Regulation of cell proliferation, both at the S- and M-phases of the cell cycle, is consistent with FoxM1 expression; however, *FoxM1*^*−/−*^ cardiomyocytes, hepatocytes, and smooth muscle cells show normal proliferation^19^. In this study, we observed that FoxM1 expression is linked with the S-phase for both diploid (mesometrial) and polyploid (SDZ) decidual cells at the IS (Fig. 1 and Supplementary Fig. S1). However, *FoxM1* deficiency had no impact on decidual cell progression during S-phase (Figs 3C,D and 4D), indicating it is not crucial during this period. Despite this, FoxM1 may still play an important role during S-phase. As many M-phase genes are induced during S-phase and then silenced in anaphase^27^,^32^, it is possible that FoxM1 mediates an interdependent function between S- and M-phases. Interestingly, we have observed that several M-phase regulatory genes, as well as M-phase cells, were strikingly downregulated by FoxM1 deficiency in both regions (Fig. 3). However, thus far FoxM1 expression has primarily been reported in connection with malignant tumor cells, while deletion of FoxM1 is mostly detrimental to cancer progression^20^, suggesting that FoxM1 plays an essential role under malignancy as opposed to normal physiological conditions. In this regard, decidual growth has been shown to share many tumor-like features such as rapid expansion, vascularization, inflammatory reaction, immune-suppressive microenvironment, and an aberrant number of chromosome copies^4^. Consistently, it is not surprising in the present study that we found the loss of *FoxM1* significantly affects cell cycle progression with enhanced polyploidy development (Figs 3 and 4), suggesting that FoxM1 may potentially control a regulatory balance between diploid vs. polyploid cell development (Fig. 7).

It has been well recognized that decidual polyploidy develops as mono- or bi-nucleated cells due to blockage in G2/M phase of the cell cycle^3^,^7^. Here, we specifically noted that FoxM1 deficiency perturbs normal cell cycle progression resulting from increased arrest prior to cytokinesis, and leads to enhanced endoreduplication for polyploidy development, as judged by the analyses of DNA content from cells *in vivo* (Fig. 3G) and *in vitro* (Fig. 4A,B), as well as by the analyses of bi-nucleation *in vivo* (Supplementary Fig. S4). These results clearly document that the loss of FoxM1 promotes transition from diploid into polyploid cells. Polyploid decidual cells predominantly constitute the development of SDZ, so the overall increase in polyploid (both mono- and bi-nucleated) cells could explain the expansion of SDZ at the site of implantation. The decrease in IS weight (Fig. 2B,C) and smaller cross section (Fig. 3A) of *FoxM1*^*d/d*^ mice suggest that the net effect of relative expansion of SDZ is inhibition of overall decidual growth. The increase in both mono- and bi-nucleated polyploid cells has been reported in breast cancer or pancreatic cells after suppression of *FoxM1*^27^,^33^,^34^,^35^, and this phenomenon appears to be common with deficiency of other mitotic regulators, such as Cdh1 and Plk1^36^,^37^. It is known that the cell cycle regulatory factors Ccnb1 (also known as cyclin B) and Cenpf possess diverse roles in M phase, such as chromosome segregation and cytokinesis^38^,^39^. The loss of *FoxM1* has been shown to cause pleiotropic cell-cycle defects, including delay in G2, aberration in chromosome segregation, and frequent failure of cytokinesis^27^. Since, both *Ccnb1* and *Cenpf* were affected by FoxM1 deficiency at the site of implantation and *in vitro* decidualization (Figs 3E and 4C), we suggest their downregulation plays a major contribution to cease cytokinesis, but enhance polyploidy and bi-nucleation in *FoxM1*^*d/d*^ mice as compared to control. In this regard, it is worth mentioning that the accelerated entry into S-phase with increased polyploidization and failure of mitosis has also been shown in *FoxM1* null fetal cardiomyocytes^40^ and hepatocytes^41^. In addition, it is worth mentioning that the size of polyploid cells in *FoxM1*^*d/d*^ mice did not reveal any significant difference as compared to *FoxM1*^*f/f*^ mice, although the size of uterine polyploid cells are much larger than that of diploid cell^6^,^31^,^42^. Therefore, we believe that the expansion of SDZ as revealed in our study was not caused by the change in cell size, but rather by increase in polyploid cell population.

The development of decidual polyploidy has been shown to be beneficial for the support of implantation progression and successful pregnancy. For example, decidua with insufficient polyploidy is inhibitory for implantation progression, as shown by deletion of *DEDD*, *Hoxa10*, or *Ccnd3* in mice^4^,^10^,^18^. However, over-amplification of decidual polyploid cells is also detrimental to the aspect of implantation, as shown by *Trp53* null mice^26^. In the present study, the increased accumulation of polyploidy (both mono- and bi-nucleation) in *FoxM1* null mice also resulted in defects, as evidenced by insufficient growth of embryo implantation and lower decidual tissue weight. Therefore, we suggest that enhancement of polyploidy, but lack of complete mitosis in *FoxM1* null cells, could reduce the pool of diploid cells, which will severely affect the repertoire of diploid cell proliferation and further decidual growth, which in turn leads to failed implantation sites in severe cases. Thus, balanced progression of proliferation and polyploidy development appears to be critical to support formation of functional decidua in implantation. Together, either deficiency or overabundance of polyploidy is detrimental to successful pregnancy.

Previously, it has been shown that the increase in decidual polyploidy at the site of implantation is associated with enhanced decidual senescence, as in the case of *Trp53* null mice^26^, although we did not see any alteration in the expression of *Trp53* or decidual senescence at the IS for *FoxM1* null vs control mice. Previously, studies have shown that enhanced polyploidy due to suppression of *FoxM1* could lead to cell death by mitotic catastrophe^33^, suggesting polyploidy mechanism limits the lifespan of cells. However, based on our analyses by immunofluorescence studies using cleaved caspase 3, a well-established proapoptotic cell marker^43^, on sections of implantation sites between *FoxM1*^*d/d*^ and *FoxM1*^*f/f*^ mice, did not reveal any increase in apoptosis in the decidual bed by *FoxM1* deficiency, as compared to control (Supplementary Fig. S6). Apoptosis is barely detected by TUNEL or other assays in normal decidual bed as previously reported by us^25^ and others^44^.

Previously, in an induced pneumonia model, expression of *FoxM1* in the knockout mice restored the proliferation and trans-differentiation defects of alveolar epithelium progenitor cells^45^. The regulatory function of FoxM1 at G2-M phase was also been rescued by its steady expression in neural plate cells^46^. Similarly, it will be interesting to study the rescue experiments whether *FoxM1* overexpression reverses the defects with enhanced cell cycle arrest prior to mitosis with aberrantly upregulated polyploidy during decidualization, which will be the subject of research in future.

FoxM1 is highly expressed in polyploid decidual cells, although its expression also revealed in mesometrial decidual cells (Fig. 1). The periimplantation uterine expression of FoxM1 appeared to be similar with Hoxa10 or cyclin D3 during decidual progression^3^,^14^,^17^. For example, all three genes exhibit weak to moderate expression on D4 in uterine stroma, but are markedly induced in decidualizing stromal cells from D5 through D8^3^,^15^,^17^,^47^, suggesting these genes may have a regulatory connection. Consistently, we observed regulation of FoxM1 gene transcription or its nuclear protein translocation in polyploid cells by *Hoxa10* or *Ccnd3* deficiency, respectively.

*Hoxa10* mutant mice are sterile due to failure of decidualization and implantation^15^, and these defects have been primarily implicated with impaired progesterone signaling for stromal cell proliferation^14^ and differentiation^13^, loss of regional decidualization^13^ and polyploidy development^18^, as well as aberrant upregulation of cell cycle inhibitory genes (*Cdkn1c, Cdkn2b, Ccng1*, and *Ccng2*)^25^,^48^ and downregulation of growth promoting cyclin (*Ccnd3/cyclin D3*)^18^. In this study, we observed that Hoxa10 directly binds to the promoter of *FoxM1* (Fig. 5), identifying as Hoxa10 downstream transcriptional target mediating cell cycle regulatory functions during decidualization. In this regard, it should be noted that *Hoxa10*^*−/−*^ mice show severe decidualization defects, as compared to *Ccnd3*^*−/−*^ or *FoxM1*^*d/d*^ mice, indicating Hoxa10 plays a pivotal upstream role for the expression of genes which effect cell proliferation and differentiation in the decidual bed. Although we noted that the loss of *Hoxa10* results in reduction to the half of FoxM1 protein levels than control (WT) (Fig. 5G), our analysis revealed that the phenotype of *FoxM1*^*f/d*^ mice, as compared to *FoxM1*^*f/f*^ (control), was normal in terms of litter size or uterine morphology, histology, and weight of implantation sites.

Cyclin D3 specifically exhibits upregulated expression during decidualization^3^,^17^ and the loss of *Ccnd3* in mice showed decidualization defects and failure of polyploidy development^4^,^31^, which could be mediated through compromised DNA synthesis^49^. We showed that the *Ccnd3* mutant mice exhibits loss of nuclear FoxM1 localization in polyploid cells (Fig. 6), while the loss of *FoxM1* showed a greater induction of polyploidy (Figs 3 and 4) and no effect on *Ccnd3* expression, indicating that cyclin D3 acting upstream for nuclear FoxM1 during decidualization, and these two regulators may participate in a feed-back loop to control appropriate polyploidy levels. Studies have shown that cyclinD3/cdk4/6 activity can target FoxM1 phosphorylation in cancer cell lines^50^. Consistent to the above notion, pharmacological inhibition of cdk4/6 kinase activity *in vitro* causes inhibition of nuclear FoxM1 translocation and loss of polyploidy, indicating nuclear FoxM1, acting downstream of cyclin D3-cdk4/6 activity. We tested the possibility of interaction between cyclin D3 and FoxM1 using immunoprecipitation with either antibody followed by immunoblotting with both antibodies. However, we were unable to see any interaction between cyclin D3 and FoxM1. Collectively, we suggest that cyclin D3 contributes to the nuclear localization of FoxM1 through cyclin D3/cdk4/6 kinase dependent pathway, without any direct protein-protein interaction between cyclin D3 and FoxM1.

Cyclin D3 is known to act only during G1 phase, while FoxM1 functions in later phases of the cell cycle, thus lacking cyclin D3 elicits defects at earlier stage than FoxM1 deficiency. Moreover, studies have shown that delay in DNA synthesis affects both cell proliferation and polyploidization^49^. Indeed, our previous studies have shown that suppression of cyclin D3 lowers the level of decidual polyploidy^31^ and overexpression of cyclin D3 rescue the defects of polyploidization in *Hoxa10* null stromal cells^18^. In contrast, the defects of FoxM1 deficiency mainly occurred during M phase, without affecting normal DNA synthesis (Figs 3C,D and 4D). Thus, this may explain further why cyclin D3-deficient mice reveal shrinkage of SDZ that is opposite to that observed in FoxM1-deficient uterus.

Subcellular distribution of FoxM1 is known to be regulated by diverse post-translational mechanisms. For example, ERK1/2 initiated phosphorylation promotes FoxM1 translocation to the nucleus^51^. SUMO1-mediated SUMOylation promotes FoxM1 translocation to the cytoplasm with inhibition of its activity and enhances ubiquitination and degradation^52^. SUMO2-mediated SUMOylation helps increase FoxM1 activity and nuclear localization^53^. The mechanism by which cyclin D3-cdk4/6 controls FoxM1 nuclear translocation in decidual cells is still not known. In this regard, it is worth mentioning that we were unable to detect modification of FoxM1 protein by SUMOylation or ubiquitination in *Ccnd3* null or cyclin D-cdk4/6 pathway inhibited cells. In our study, the immunoblotting was performed in reducing condition to prevent formation of non-specific protein aggregates, but without affecting true protein modifications. Additionally, the inclusion of 1 mM DTT to lysis buffer or β-mercaptoethanol to sample buffer, should not affect SUMO or ubiquitin mediated modifications under reducing condition, as previously reported^54^,^55^. Moreover, we were unable to ascertain phosphorylation status of FoxM1 *in vivo,* because availability of phosphorylated site-specific FoxM1 antibodies is currently limited. Overall, FoxM1 is an important effector acts downstream of cyclin D3 in controlling nuclear activity for gene regulation (Figs 3E and 4C,F).

Although loss of FoxM1 can lead to defects in decidual development, certain embryo implantation sites are able to overcome these defects and survive through term labor. We do not know the precise reason why certain sites in the mutant uterus can accommodate embryos while others cannot, but this is not an uncommon phenomenon in implantation^4^. To our knowledge there is no report to suggest that FoxM1 can have a redundancy with other Forkhead box proteins. In this regard, it is interesting to note that FoxO proteins (FoxO1/FoxO1A and FoxO3a) have been shown to play roles in human decidualization^56^,^57^, although their roles in mouse decidualization remain unknown.

In conclusion, we have presented novel comprehensive evidence supporting the hypothesis that the mesometrial decidual bed (a presumptive site for placentation) is developmentally interlinked with the appropriate progression of the SDZ at the site of implantation. In this regard, we have provided evidence that FoxM1 is regulated during decidualization and necessary for appropriate control of stromal cell cytokinesis and polyploidy development during decidual progression. Also, Hoxa10 and cyclin D3 utilize FoxM1 as a downstream effector in this process.

## Methods

### Animals

*Hoxa10*^*−/−* ^15, *Ccnd3*^*−/−* ^58, *PR-Cre* (*Pgr*^*Cre/+*^)^22^, and *FoxM1*^*f/f*^ lines^28^ were generated as previously described. *FoxM1* deleted (*FoxM1*^d/d^) mice were generated by mating *FoxM1*^f/f^ mice with *PR-Cre* mice^22^. Induction of pregnancy, artificial decidualization, tissue collection, and examination of implantation sites in early pregnancy were performed as previously described^13^,^18^. All mice were housed in the animal care facility at Cincinnati Children’s Hospital Medical Center according to National Institutes of Health and institutional guidelines for the use of laboratory animals. All protocols were approved by the Institutional Animal Care and Use Committee (IACUC) (Approval number: IACUC2013-0059).

### *In situ* hybridization

The procedures for ^35^S-labeled antisense or sense cRNA probes and *in situ* hybridization were previously described^59^.

### Reverse transcription (RT) and quantitative PCR (qPCR)

RNA (0.5–2 μg) was primed with random-hexamers in a volume of 20 μl and reverse transcribed into cDNA with MMLV Reverse Transcriptase (Promega, cat# M1701). Comparative cDNAs were quantitatively analyzed by real-time PCR using Fast SYBR® Green Master Mix reagent (Life Tech, Grand Island, NY, cat# 4385610) and the ABI StepOnePlus System (Applied Biosystems, Foster City, CA) according to the manufacturer’s instructions. All samples were run for 40 cycles of 3 sec at 95 °C and 30 sec at 60 °C, followed by melting curve stage consisting of temperature increase at 0.3 °C per min to 95 °C. Melting curves for all products showed single peaks. The relative target gene expression was quantified by the ΔΔCt method^60^, using *ribosomal protein l7 (Rpl7*, housekeeping gene) for normalization. Primer sequence information for analyzed genes is listed in Supplementary Table S1.

### Immunohistochemistry and immunofluorescence

Sections of paraffin-embedded neutralized buffered formalin fixed tissue were used for Ni-DAB (FoxM1) or DAB (other antibodies) colorimetric immunohistochemistry (IHC), while paraformaldehyde fixed frozen tissue sections or cells grown on coverslips were subjected to immunofluorescence (IF). Antibodies include rabbit anti-FoxM1 (1:1000 for IHC or 1:500 for IF; Santa Cruz, cat# sc-502), rabbit anti-cyclin D3 (1:500; Santa Cruz), rabbit anti-Ki67 (1:500; Thermo), rat anti-BrdU (1:200; Abcam), rat anti-phosphorylated histone H3 (Ser 10) (1: 1000; Millipore), rabbit anti-PR (1:300; Santa Cruz), rabbit anti-ERα (1:300; Santa Cruz), and rabbit anti-cleaved caspase 3 (1:300 for IF; Santa Cruz). Counting of immunostaining-positive cells were determined using the Image J program available at http://imagej.nih.gov/ij (NIH, USA), and the analyses were based on examination of serial sections for at least 5–6 IS samples collected from 3–5 different mice for each group.

### Chromatin immunoprecipitation

This procedure was followed with some modifications of previously described methods^61^. Deciduoma tissues collected after 3 days of induction were minced into small pieces and incubated in 1% paraformaldehyde/ PBS for 15 min to crosslink DNA and proteins. After termination of reaction by 0.125 M glycine, tissues were grinded in 1% SDS Tris buffer, followed by sonication (at 50% output) for 8 pulses at 10 sec each using Sonic Dismembrator (Fisher Scientific). Sonicated chromatin was incubated with goat anti-Hoxa10 (1:50; Santa Cruz), mouse anti-RNA polymerase II (1:200; Millipore) or corresponding IgGs on a rotator overnight at 4 °C. The immunoprecipitated chromatins were retrieved by binding to protein G-coupled Dynabeads (Invitrogen). After low salt, high salt, LiCl and TE buffer washing, DNA was recovered after elution, RNase and proteinase K digestion and column based purification as described commercially (Qiagen). DNA was analyzed by qPCR and binding levels were determined as percentage against the input samples and expressed as fold enrichment against IgG. Primer sequence information for analyzed genes is listed in Supplementary Table S2.

### Preparation of cellular extracts and western blotting

Whole uterine tissues on D4 or ISs on D5-D8 were used for protein extraction. For subcellular fraction analysis, deciduomal tissues separated from myometrium were divided into mesometrial and SDZ (lateral + antimesometrial) and minced into small pieces, incubated with hypotension buffer (10 mM HEPES pH 8.0, 1.5 mM MgCl2, 10 mM KCl, 0.1 mM EDTA, 1 mM DTT), then passed successively through 18, 22, and 25 gauge needles followed by 40 μm cell strainers and centrifuge to separate the nuclear and cytoplasmic fractions. Protein was extracted with RIPA buffer and subjected to western blotting as described previously^3^. All antibodies were purchased from Santa Cruz unless specifically stated and they include rabbit anti-FoxM1 (1:2000), goat anti-Actin (1:1000), rabbit anti-Lamin A/C (1: 1000), mouse anti-tubulin α (1: 5000), rabbit anti-cyclin D3 (1: 1000), goat anti-Hoxa10 (1: 1000), and rabbit anti-cyclin B1 (1: 500).

### Flow cytometry analysis

This procedure was followed as described previously^6^. Decidual cells were isolated from implantation sites after removal of embryos and passed through various gauges of needles to prepare single-cell suspensions using Cycle Test Plus DNA Reagent Kit (BD). Cultured uterine stromal/decidual cells were fixed in 70% ethanol, treated with RNase A (500 μg/ml) for 30 min at 37 °C, and then stained with propidium iodide (PI, 50 μg/ml). Analyses of DNA content was done by flow cytometry (BD FACSCanto II). At least 5,000 to 10,000 cells were subjected for each analysis.

### Cell culture and treatment

The procedures for uterine stromal cell isolation, culture and induction of decidualization were followed as we previously described^31^. In brief, stroma cells at around 50% confluence were cultured in DMEM/F-12 (1:1) medium containing 1% charcoal-stripped-fetal bovine serum (CS-FBS), and decidualization was induced by addition of 1 μM P4, 10 nM E2 and 10 ng/ml HB-EGF. Cdk4/6 inhibitor PD0332991 (10 μM; Sigma) and dimethyl sulfoxide (DMSO, 0.1%), a vehicle control, were added to cells at the time of decidual stimulation and cells were analyzed after 48 h. For cell cycle proliferation analysis by flow cytometry, seeded stromal cells were starved for synchronization in absence of FBS (overnight), then cultured in 1% charcoal stripped (CS)-FBS containing DMEM/F-12 (1:1), and cells were collected at 12, 16, 20, and 24 h.

### Measurement of serum estradiol-17β and progesterone levels

Sera were collected on day 8 of pregnancy, and estradiol-17β and progesterone levels were measured by EIA kits (Cayman Chemical).

## Additional Information

**How to cite this article**: Gao, F. *et al.* Control of regional decidualization in implantation: Role of FoxM1 downstream of Hoxa10 and cyclin D3. *Sci. Rep.* **5**, 13863; doi: 10.1038/srep13863 (2015).

## Supplementary Material

Supplementary Information
