# Supplementary material for: Control of regional decidualization in implantation: Role of FoxM1 downstream of Hoxa10 and cyclin D3
Source: Sci Rep. 2015 Sep 9;5:13863. doi: 10.1038/srep13863 (PMC4563553; doi:10.1038/srep13863)
Supplement: Supplementary Information [file srep13863-s1.pdf]

Control of regional decidualization in implantation: Role of FoxM1 downstream of Hoxa10 and cyclin D3

Fei Gao[1,2], Fenghua Bian[1,2], Xinghong Ma[1,2,4], Vladimir V. Kalinichenko[2,3], and Sanjoy K. Das[1,2]*

[1] Division of Reproductive Sciences, Cincinnati Children's Hospital Medical Center, University of Cincinnati College of Medicine, Cincinnati, OH 45229, USA

[2] Perinatal Institute, Cincinnati Children's Hospital Medical Center, University of Cincinnati College of Medicine, Cincinnati, OH 45229, USA

[3] Division of Pulmonary Biology, Cincinnati Children's Hospital Medical Center, University of Cincinnati College of Medicine, Cincinnati, OH 45229, USA

[4] College of Life Science, Northeast Agricultural University, Harbin, China

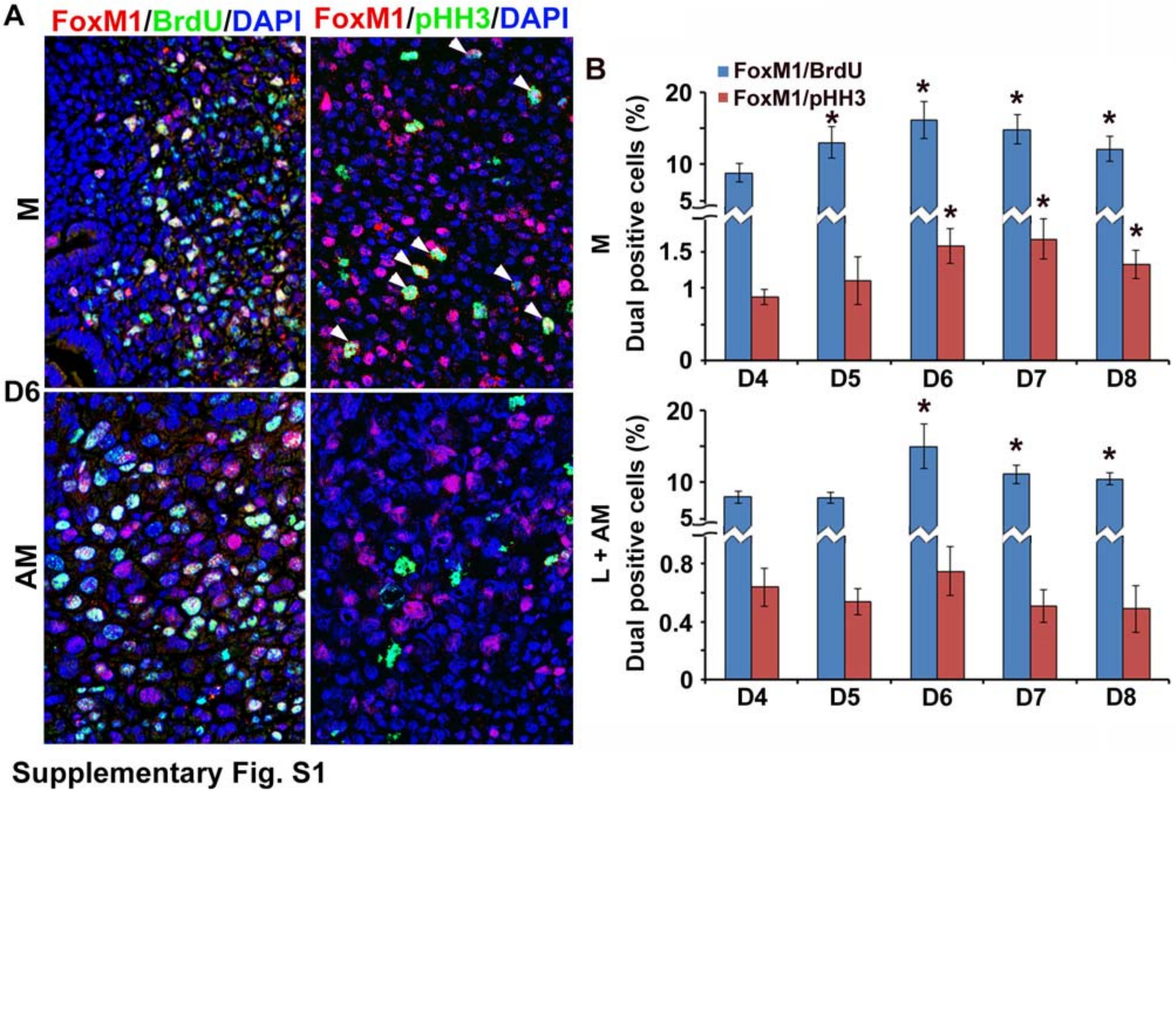

Supplementary Fig. S1

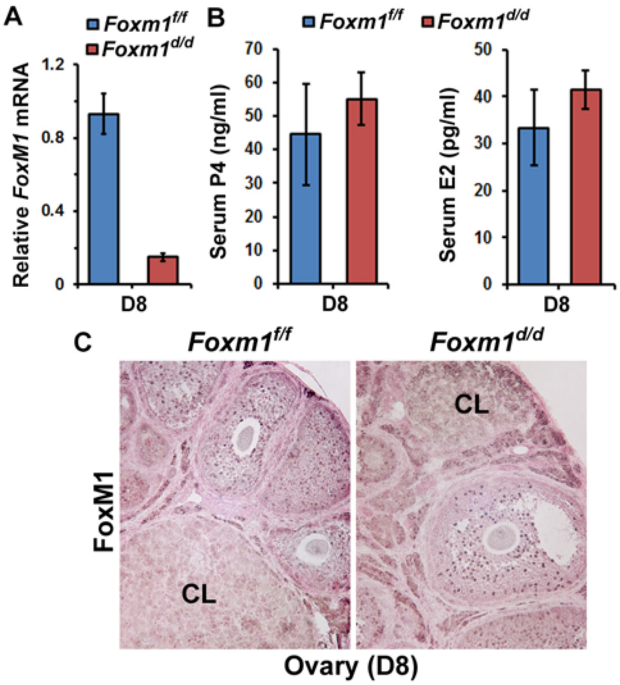

Supplementary Fig. S2

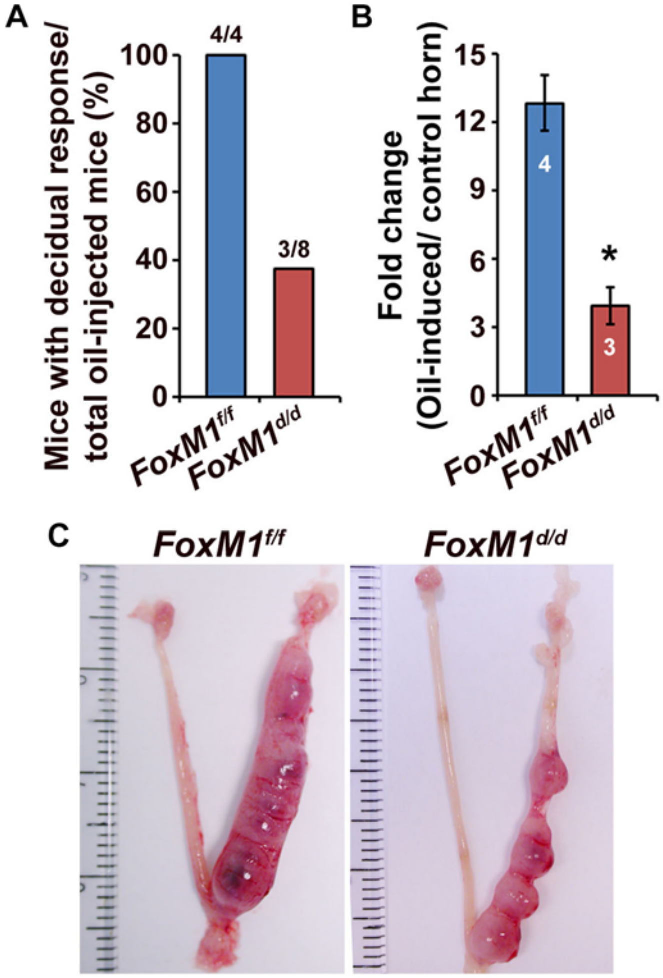

Supplementary Fig. S3

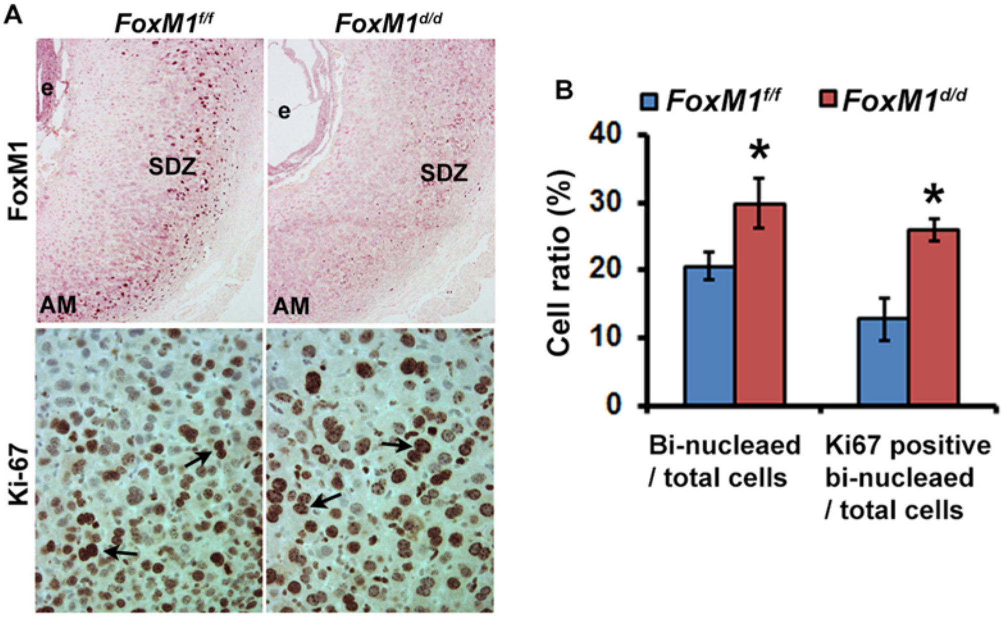

Supplementary Fig. S4

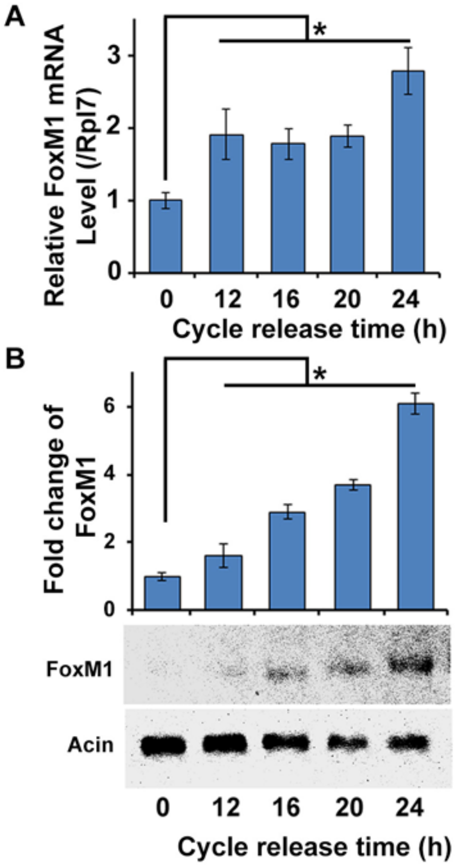

Supplementary Fig. S5

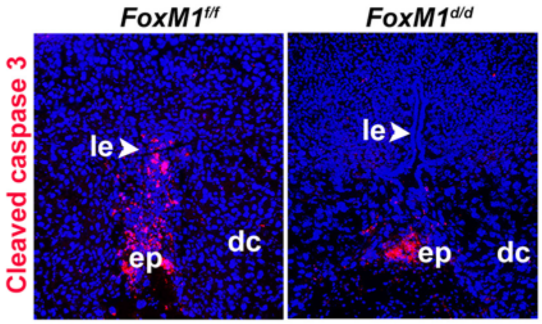

Supplementary Fig. S6

Supplementary Figure legends

Supplementary Fig. S1. Immunofluorescence analyses of FoxM1 in peri-implantation uteri in conjunction with cell cycle phase-specific markers. A. Localization of FoxM1 (red) together with BrdU (green) (for S-phase) or pHH3 (green) (for M-phase) is shown for D6 in the left or right columns, respectively. DAPI was used for nuclear staining. Magnifications are at 600X. **B**. Quantitative analyses of dual positive FoxM1/BrdU or FoxM1/pHH3 cells (%) in the M and L+AM locations on D4 and D5-8 IS. *, $p < 0.001$.

Supplementary Fig. S2. Analyses of uterine *FoxM1* deletion, in conjunction with ovary function, for *FoxM1*$^{d/d}$ vs. *FoxM1*$^{f/f}$ mice. A. Quantitative RT-PCR analysis of *FoxM1* at D8 IS. **B**. Analyses of serum levels of progesterone (P4) and estrogen (E2) on D8. **C**. Immunostaining of FoxM1 in ovaries on D8. CL, corpus luteum.

Supplementary Fig. S3. **The experimental decidualization response between *FoxM1*$^{d/d}$ vs. *FoxM1*$^{f/f}$ mice. A**. The outcome of decidualization response after infusion of sesame oil (20 μl) intraluminally in one horn on D4 of pseudopregnancy for control and null mice. The contralateral horn served as control. Mice were examined on D8. Numbers above the bars indicate the number of mice with decidual response compared to the total number of mice examined. **B**. Fold increase in uterine weights (infused vs. non-infused) after intraluminal oil infusion and examined on D8. *, $p < 0.001$. **C**. Uterine morphology after induction of decidualization on D8.

Supplementary Fig. S4. Analysis of bi-nucleation at the IS between *FoxM1*$^{d/d}$ vs. *FoxM1*$^{f/f}$ mice on D8. A. Immunostaining localization of FoxM1 and Ki67. Arrows indicate bi-nucleated cells. **B**. Quantitative analyses of Ki67-positive and hematoxylin-stained bi-nucleated cells in SDZ. *, $p < 0.001$.

Supplementary Fig. S5. FoxM1 expression both at mRNA (A) and protein (B) levels, during cell cycle phase progression for 0-24 h, following the cycle release. **A**. Quantitative RT-PCR. **B**. Western blotting. *, $p < 0.05$.

Supplementary Fig. S6. Expression of cleaved caspase 3 at the IS between *FoxM1*$^{d/d}$ vs. *FoxM1*$^{f/f}$ mice on D8. Immunofluorescence localization. DAPI was used for nuclear staining. le, luminal epithelium; ep, ectoplacental cone; dc, decidual cells;

Supplementary Table S1. Sequence of primers used for qPCR analysis.

Gene	Forward primer	Reverse primer
Foxm1	cacttggattgaggaccactt	gtcgtttctgctgtgattcc
Rpl7	gcagatgtaccgcactgagattc	acctttgggcttactccattgata
Bmp2	agatctgtaccgcaggcact	aagttcctccacggcttctt
Hoxa10	cgcctagagatcagccgtag	tcaggaagcgaaaagacgtt
Ccnd3	cgagcctcctacttccagtg	agccagagggaagacatcct
Trp53	tggaagactccagtgggaac	tcttctgtacggcggtctct
Tdo2	tgaaaggcctggaagaagaa	cgcttctcatcaaacaagca
Ccna1	tcttccttccttggttgctg	acttctccctgattgcttgc
Ccna2	acagagctggcctgagtcat	ttgactgttgggcatgttgt
Ccne1	cctccaaagttgcaccagtt	cacccgtgtcgttgacatag
Ccne2	tctgtgcattctagccatcg	acaaaaggcaccatccagtc
E2f1	gaggctggatctggagactg	gaagcgtttggtggtcagat
Cdk1	ctgggcactcctaacaacgaag	tccaagccgttctcgtccag
Cdk2	ccccagaacctgcttatcaa	gcagcccagaagaatttcag
Cdk4	ggccctcaagagtgtgagag	catcagccgtacaacattgg
Cdk6	agaagtcctgctccagtcca	cacgtctgaacttccacgaa
Cdc25a	gggaagcatcaggatttgaa	cacccttgatgtgacctcct
Cdc25b	tccttaccagtgaggctgct	tcggtagcctgcttcagttt
Cks1b	gctggtacctgctttgcttc	cacgtcagcaaattcacacc
Gas1	accgattcattcctgtgctc	cagaatggtggcaggaaaat
Nek2	ccgagagcctgatgaagaac	gcggtgttctctttgctttc
Aurkb	tcgctgttgtttccctctct	ggctccttccgtaggactct
Birc5	ctgatttggcccagtgtttt	caggggagtgctttctatgc
Plk1	gtgatggcacggagtcctat	cagcaggtgctcactcatgt
Cenpf	cgtgaaagcgactcattgaa	tgccagctcttggttttctt
Skp2	aactgcgcctatttcaccac	gggcttttgcagagtcagtc
Ccnb1	tggactacgacatggtgcat	caggtgctgcataacaggaa
Ccnb2	acccacagcctctgtgaaac	cttgcagagcagagcatcag
Cdkn1a	gttccttgccacttcttac	actgcttcactgtcatcc
Cdkn1b	agcgtttcttcattgcctgt	cacaaaacatgccactttgg
Cdkn1c	ggagcaggacgagaatcaag	gttctcctgcgcagttctct

Supplementary Table S2. Sequence of primers used for ChIP-PCR analysis of Hoxa10 binding on *FoxM1* gene.

Primers pair	Forward primer	Reverse primer
1	tttcctaccccgaccttacc	tccagactaggcttccctga
2	tgcagtaaatcatgcctcca	tccagaccaaccgaggatag
3	ctagctctctgaccccatcg	atggctgccgagtttttaga
4	ctccccgtccttaaacttcc	taatccatgcaaatccagca
5	ttgaggcacagtggtacagc	gtgaccttcctgctttctgc
6	agctgggcacatcaccttag	aaataggcaaaggctgagca
7	cctgcctcctgtctcatagc	cgcagcctcctgtgataact
8	tggcaaacgtcaaaagagtg	gcctggctatgctaaagtgg
9	ctaacaggtggaggcaggag	ctgatggctcacagcaatgt
10	gctgcaactggtagctttcc	ggctgagacaaactgcatga
